# NK Cell-Mediated Eradication of Ovarian Cancer Cells with a Novel Chimeric Antigen Receptor Directed against CD44

**DOI:** 10.3390/biomedicines9101339

**Published:** 2021-09-28

**Authors:** Rüdiger Klapdor, Shuo Wang, Michael A. Morgan, Katharina Zimmermann, Jens Hachenberg, Hildegard Büning, Thilo Dörk, Peter Hillemanns, Axel Schambach

**Affiliations:** 1 Department of Gynecology and Obstetrics, Hannover Medical School, 30625 Hannover, Germany; wangshuo1022@gmail.com (S.W.); hachenberg.jens@mh-hannover.de (J.H.); doerk.thilo@mh-hannover.de (T.D.); Hillemanns.Peter@mh-hannover.de (P.H.); 2 Institute for Experimental Hematology, Hannover Medical School, 30625 Hannover, Germany; morgan.michael@mh-hannover.de (M.A.M.); zimmermann.katharina@mh-hannover.de (K.Z.); buening.hildegard@mh-hannover.de (H.B.); 3 REBIRTH Center for Translational Regenerative Medicine, Hannover Medical School, 30625 Hannover, Germany; 4 Comprehensive Cancer Center Niedersachsen, CCC Hannover, Hannover Medical School, 30625 Hannover, Germany; 5 Division of Hematology/Oncology, Boston Children’s Hospital, Harvard Medical School, Boston, MA 02115, USA

**Keywords:** chimeric antigen receptor, ovarian cancer, CD44, adoptive immunotherapy, NK cells

## Abstract

Ovarian cancer is the most common cause of gynecological cancer-related death in the developed world. Disease recurrence and chemoresistance are major causes of poor survival rates in ovarian cancer patients. Ovarian cancer stem cells (CSCs) were shown to represent a source of tumor recurrence owing to the high resistance to chemotherapy and enhanced tumorigenicity. Chimeric antigen receptor (CAR)-based adoptive immunotherapy represents a promising strategy to reduce the risk for recurrent disease. In this study, we developed a codon-optimized third-generation CAR to specifically target CD44, a marker widely expressed on ovarian cancer cells and associated with CSC-like properties and intraperitoneal tumor spread. We equipped NK-92 cells with the anti-CD44 CAR (CD44NK) and an anti-CD19 control CAR (CD19NK) using lentiviral SIN vectors. Compared to CD19NK and untransduced NK-92 cells, CD44NK showed potent and specific cytotoxic activity against CD44-positive ovarian cancer cell lines (SKOV3 and OVCAR3) and primary ovarian cancer cells harvested from ascites. In contrast, CD44NK had less cytotoxic activity against CD44-negative A2780 cells. Specific activation of engineered NK cells was also demonstrated by interferon-γ (IFNγ) secretion assays. Furthermore, CD44NK cells still demonstrated cytotoxic activity under cisplatin treatment. Most importantly, the simultaneous treatment with CD44NK and cisplatin showed higher anti-tumor activity than sequential treatment.

## 1. Introduction

Ovarian cancer remains the most lethal among gynecological cancers [1]. Unfortunately, because localized ovarian cancer is generally asymptomatic and screening tests have not been successfully implemented, 75% of patients present with advanced tumor stages (FIGO stages III–IV) at the time of diagnosis [2]. Therefore, there is an urgent need for the development of novel and effective therapeutic strategies. It is well-known that the immune system plays an important role in monitoring tumor development and progression [3]. Compelling clinical evidence shows that the presence of tumor-infiltrating lymphocytes (TILs) is associated with a favorable prognosis in ovarian cancer [4,5,6,7]. These studies suggest that the immune system plays a substantial role in controlling ovarian cancer progression and that immunotherapy might present an effective therapeutic option. 

Chimeric antigen receptor (CAR)-modified T and natural killer (NK) cells have gained recent attention in ovarian cancer treatment [8,9]. CARs are synthetic receptors that consist of a single-chain variable fragment (scFv) for antigen recognition, a transmembrane domain and a cytoplasmic domain to activate the NK cells. Second and third-generation CARs were developed, which additionally contain one or two co-stimulatory domains, such as those derived from CD28 and/or 4-1BB, to improve survival and proliferation of the effector cells [10,11].

Recurrence followed by chemoresistance is one of the main contributing factors to the poor prognosis in ovarian cancer patients. Many researchers have demonstrated the existence of ovarian cancer stem cells (CSCs) with increased tumorigenicity, differentiating capacity and chemoresistance [12,13]. Therefore, targeted therapy against CSCs has emerged as a promising strategy in combination with conventional chemotherapy in ovarian cancer. CD44 expression has been shown to be associated with CSC-like properties in a variety of tumors [14,15], including ovarian cancer [16]. In addition, CD44 is known to be the major receptor for hyaluronan (HA), which is implicated in cell–cell and cell–matrix interactions and is associated with the promotion of cancer metastasis [17,18]. The binding of HA to CD44 was shown to mediate ovarian cancer cell adhesion to peritoneal mesothelial cells [19,20]. Collectively, CD44 could be a promising target molecule for immunotherapy in ovarian cancer.

In this study, we developed a novel CD44-specific third-generation CAR and analyzed its anti-tumor activity in ovarian cancer cell lines and primary patient-derived ovarian cancer cells. To maximize the benefits of anti-CD44 CAR expressing NK cells, the cytotoxicity was also evaluated in combination with chemotherapy.

## 2. Material and Methods

### 2.1. Cell Lines

Human embryonic kidney 293T cells (HEK-293T, ATCC CRL-3216) were used for lentiviral vector production and grown in Dulbecco’s modified Eagle’s medium (Biochrom, Berlin, Germany) supplemented with 10% heat inactivated FBS, 100 U/mL penicillin, 100 mg/mL streptomycin, and 10 mmol/L HEPES. NK-92 cells were cultured in RPMI-1640 medium (Sigma-Aldrich, Steinheim, Germany) supplemented with stable L-Glutamine, 10% FBS, 100 U/mL penicillin, 100 mg/mL streptomycin, and 200 IU/mL IL-2 (Proleukin S; Novartis Pharma GmbH, Nürnberg, Germany). Ovarian cancer cell lines A2780, SKOV3, and OVCAR3 were stably equipped with EGFP by lentiviral transduction and cultured in RPMI-1640 medium supplemented with stable L-Glutamine, 10% FBS, 100 U/mL penicillin, and 100 mg/mL streptomycin. After obtaining informed consent, primary ovarian cancer cells (P1, P2 and P3) were harvested from sequential ascites samples of an ovarian cancer patient and cultured in the same condition as ovarian cancer cell lines in low-attachment flasks (Corning, Wiesbaden, Germany).

#### Cloning of Vectors and Lentiviral Vector Production

The sequence of CD44 scFv was selected from a fully human anti-CD44 monoclonal antibody (mAb) called PF-03475952 [21]. All sequences underwent optimization of codon usage and GC content to increase the efficiency of transcriptional processing and protein expression. The recognition sequences of restriction enzymes (AgeI, NotI, SalI, and BsrgI) were removed, and then the DNA synthesis was performed by GeneArt (Thermo Fisher, Regensburg, Germany). Our anti-CD44 CAR was generated from an already published third-generation anti-CD19 CAR [22] by replacing the scFv fragment. This anti-CD19 CAR contains CD28, 4-1BB and CD3ζ domains and served as a control in further experiments. After producing a third-generation lentiviral SIN vector, an internal ribosomal entry site (IRES)-driven dTomato expression cassette was inserted between SalI sites to allow co-expression and facilitate detection of transduced cells.

To produce lentiviral vectors, HEK-293T cells were transfected using a calcium phosphate method. HEK-293T cells (5 × 10^6^) were seeded in 10 cm dishes and cultured overnight. The following plasmids for each dish were mixed and diluted in water with the desired volume: 12 µg of the vector plasmid, 12 µg of pcDNA3.GP.4 × CTE (gag/pol), 5 µg of pRSV-Rev and 2 µg of RD114/TR envelope plasmids. The purified lentiviral packaging plasmids were purchased from Plasmid Factory (Bielefeld, Germany). Viral supernatants were harvested 36 h after transfection, filtered through MillexGP 0.22 µm filters (Millipore, Schwalbach, Germany), concentrated via ultracentrifugation, and stored at –80 °C until use.

### 2.2. Transduction of NK Cells

NK-92 cells were equipped with CAR constructs by Retronectin-assisted transduction. Briefly, 48-well plates were coated with Retronectin (Takara, Shiga, Otsu, Japan) (210 μL of 24 µg/mL in PBS per well) overnight at 4 °C or 2 h at room temperature. Retronectin was then removed. The wells were blocked with sterile-filtered PBS containing 2% BSA for 30 min at room temperature. After washing with HBSS/HEPES (Biochrom, Berlin, Germany), viral supernatants were then added into the Retronectin-precoated plates and centrifuged for 30 min at 400× *g* and 4 °C. Afterwards, 5 × 10^4^ NK-92 cells were added and incubated for 24 h. Then, the cells were transferred to uncoated plates.

### 2.3. IFNγ Release Assays

IFNγ release assays were performed in triplicate by co-culture of NK cells and target cells at an E/T ratio of 5:1 in 96-well plates in a final volume of 200 µL of NK cell media containing 200 IU/mL IL-2. After 24 h, cell fraction-free co-culture supernatants were assayed for presence of IFNγ using DuoSet Ancillary Reagent Kit (R&D Systems, Minneapolis, MN, USA), according to the manufacturer’s instructions. The average zero standard optical density (O.D.) was measured by the microplate spectrophotometer at a wavelength of 450 nm, and correction was set to 540 nm.

### 2.4. Cytotoxicity Assays

#### 2.4.1. Live Cell Imaging Using Fluorescent Microscopy

GFP expressing ovarian cancer cells were seeded in 48-well plates and cultured overnight. The effector NK cells were then added at an effector-target (E/T) ratio of 5:1. Time-lapse imaging was immediately started with temperature and gas control. Phase-contrast and fluorescent images of each position were taken every 15 min. The positions were selected and saved by the software before addition of NK cells; the acquisition focal plane was quickly set afterwards.

#### 2.4.2. Analysis of Cytotoxicity by Fluoroskan Ascent™ FL

Ovarian cancer cells were seeded in flat-bottom 96-well plates (Sarstedt, Nürmbrecht, Germany) at appropriate densities (A2780, 2 × 10^4^ cells/well; SKOV3 and OVCAR3, 1.5 × 10^4^ cells/well) and cultured overnight. NK-92 and CAR-NK-92 cells were added at an E/T ratio of 5:1 on the following day. At several time points, culture medium containing NK cells and cell debris was completely removed by inverting the plates and blotting them against clean paper towels. After adding 200 µL 5% (*w*/*v*) SDS into each well, the fluorescence intensities of GFP in the cell homogenate, which corresponds to the cell numbers, was measured at excitation 485 nm/emission 520 nm using Fluoroskan Ascent™ FL (Thermo Fisher Scientific, Waltham, MA, USA).

#### 2.4.3. Analysis of Cytotoxicity by xCELLigence

To estimate the anti-tumor activity of NK cells against unmarked primary ovarian cancer cells, the xCELLigence RTCA SP instrument (ACEA Biosciences, San Diego, CA, USA) was used according to the instructions of the supplier. To achieve equilibrium of E-Plate 96 surface (ACEA Biosciences), 100 µL of cell culture media were added to each of the 96 wells before seeding the target cells. The plates were then left in the cell culture hood for 30 min at room temperature. Afterwards, the background impedance of cell culture media was measured for calculation of the cell index value. Next, the E-Plate 96 was removed from the incubator and the desired cells were added in 50 µL medium. The cell suspension was properly prepared for the appropriate cell concentrations previously determined by a titration experiment (A2780: 2 × 10^4^; OVCAR3: 1.5 × 10^4^; SKOV3 and primary ovarian cancer cells: 1 × 10^4^). The plate was left in the culture hood for 30 min to allow the cells to settle to the bottom of the well. Then, the E-Plate 96 was reinserted, and the impedance of each well was measured every 15 min. The next day, when the cells reached the logarithmic growth phase, E-Plate 96 was removed from the incubator and effector NK cells were added in 50 µL medium at the desired E/T ratio. Then, the impedance was measured every 1 min for 8 h and every 15 min thereafter. The experiments were manually stopped 48 to 72 h after the addition of effector cells.

### 2.5. Chemotoxicity Assays

A2780 or primary ovarian cancer cells P3 were seeded in 48-well plates in the appropriate cell number. NK cells at an E/T ratio of 2:1 and cisplatin at the previously determined IC_50_ concentration (Sigma Aldrich, St. Louis, MO, USA) were added in 200 μL medium. Total incubation time was 96 h. In the sequential treatment groups, co-incubation with NK cells was performed for 24 h and cisplatin treatment for 72 h. Cells were washed twice before changing conditions to ensure no NK cells or cisplatin remained. Controls were analyzed at each step to reduce systematic bias. After treatment, all wells were washed twice and analyzed with the CellTiter 96^®^ AQueous One Solution Cell Proliferation Assay (Promega, Fitchburg, WI, USA) following the manufacturer’s protocol.

### 2.6. Statistical Analysis

Data from the experiments are expressed as means ± standard deviations. Two-way ANOVA combined with Tukey’s multiple comparisons test was used to analyze Fluoroskan results. One-way ANOVA combined with Tukey’s multiple comparisons test was used for comparison of differences among indicated groups. A *p* <0.05 was considered significant.

## 3. Results

### 3.1. Generation of a New Codon-Optimized Anti-CD44 CAR

**Figure 1:**
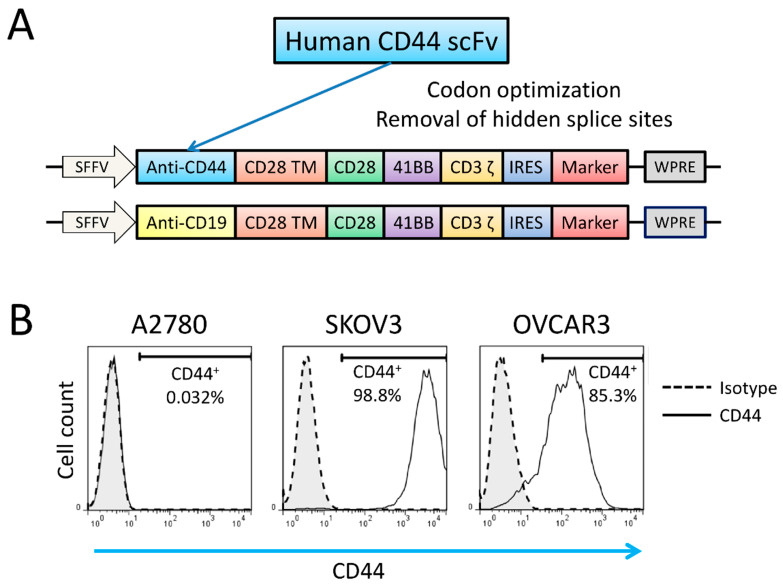
The structure of the new codon-optimized anti-CD44-CAR and antigen expression on target cells. (**A**) Schematic illustration of the modular architecture of newly developed third-generation CARs. (**B**) Flow cytometric analyses of the antigen expression of CD44 on ovarian cancer cell lines.

The CAR sequence was cloned into a third-generation lentiviral SIN vector (pRRL.PPT) [23]. After production of lentiviral vector, retronectin-mediated transduction was performed on human NK-92 cells. The design of the CAR is shown in Figure 1A.

A2780, SKOV3 and OVCAR3 cell lines were used as target cells. Surface CD44 expression levels of these human ovarian cancer cell lines were evaluated by flow cytometry. SKOV3 and OVCAR3 were shown to have high CD44 expression (98.8% and 85.3%, respectively), whereas A2780 lacked CD44 expression (Figure 1B).

### 3.2. CD44NK Cells Show Specific Cytotoxicity against Ovarian Cancer Cell Lines

**Figure 2:**
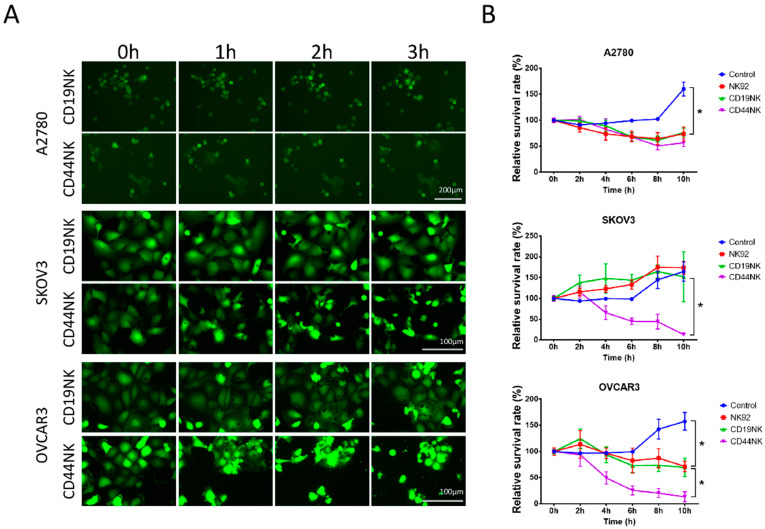
(**A**) Visualization of killing by CAR-NK-92 cells by fluorescence microscopy. A2780, SKOV3, and OVCAR3 cells were co-cultured with effector NK cells at an E/T ratio of 5:1. CD19NK cells were used as an antigen-specificity control. During the early process of death, the cells became smaller in size with condensed cytoplasm and tightly packed organelles. After cell shrinkage, massive membrane blebbing occurred, followed by separation of cell fragments into apoptotic bodies and subsequently loss of the GFP signal. (**B**) Fluoroskan results showing the killing effect of the CAR-NK-92 cells in ovarian cancer cell lines. GFP-expressing A2780, SKOV3, and OVCAR3 cells were seeded in flat-bottom 96-well plates at previously determined densities (A2780, 2 × 10^4^ cells/well; SKOV3 and OVCAR3 1.5 × 10^4^ cells/well). On the next day, NK-92 and CAR-NK-92 cells were added at the effector/target (E/T) ratio of 5:1. After removing culture medium containing NK cells and cell debris, residual attached cells were lysed with SDS, and fluorescence intensity was measured at excitation 485 nm/emission 520 nm using Fluoroskan Ascent™ FL. * indicates a significant difference calculated by two-way ANOVA, *p* < 0.05. Values represent the mean from two separate experiments each containing three samples.

To directly visualize the CAR-NK-92 cell-killing process, ovarian cancer cell lines expressing EGFP were co-cultured with CAR-NK-92 cells at an E/T ratio of 5:1. As soon as NK cells were added, a series of images were taken every 15 min by fluorescence microscopy. Figure 2A shows the morphological changes of the cell death induced by CAR-NK cells.

To quantify the cytotoxicity of CAR-NK-92 cells, a fluorescence-based cell survival assay was used. CAR-NK-92 cells and NK-92 cells were co-incubated with GFP expressing ovarian cancer cells (E/T ratio of 5:1). The GFP intensities were measured with the Fluoroskan reader every 2 h. Compared to A2780 and OVCAR3 cells cultured alone, viability of these cancer cells was significantly reduced by co-culture with untransduced NK-92 cells (*p* < 0.0001) (Figure 2B). However, the nonspecific cytotoxicity of NK-92 and control CD19NK cells did not affect the survival rate of SKOV3 cells as compared to SKOV3 cells cultured alone (*p* = 0.939). On the contrary, the survival rate in SKOV3 cells was significantly decreased by CD44NK cells (*p* < 0.0001). Significant cytotoxicity of CD44NK cells was also observed in OVCAR3 cells. No enhanced cytotoxicity of CD44NK cells was observed in CD44-negative A2780 cells. No differences in survival of all three cell lines were observed between those co-incubated with CD19NK cells or NK-92 cells. To further test the capacity of CD44NK cells to specifically eliminate target cells, a CD44-expressing lentiviral vector (co-expressing eGFP) was introduced into the CD44-negative A2780 cells. As shown in Figure S1, enforced expression of CD44 resulted in more efficient elimination of the CD44-modified A2780 cells by CD44NK cells. This was also shown by fluorescence microscopy of these co-cultures (Figure S2).

### 3.3. CD44NK Cells Specifically Kill Primary Patient-Derived Ovarian Cancer Cells

**Figure 3:**
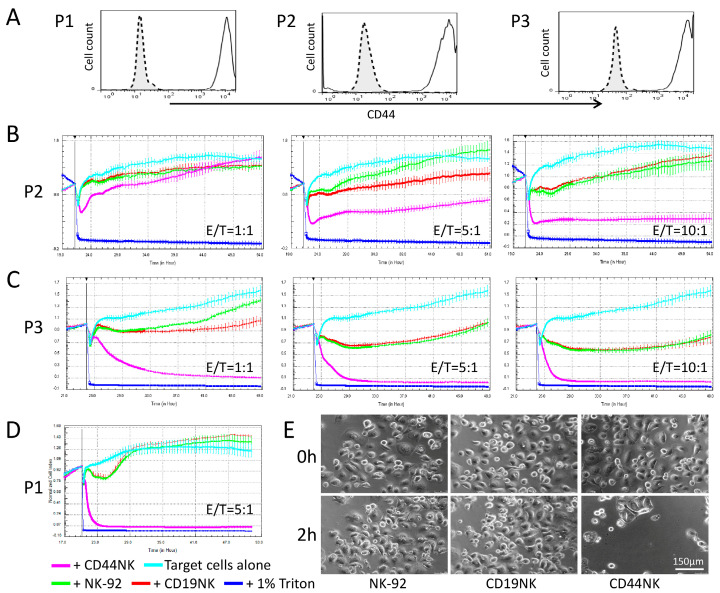
CD44NK cells specifically kill primary patient-derived ovarian cancer cells. (**A**) Flow cytometric analyses of expression levels of CD44 on three different primary ovarian cancer cell samples collected from one patient before chemotherapy (P1) or during chemotherapy (P2 and P3). Cytotoxic effects of engineered NK-92 cells on primary ovarian cancer cells P2 (**B**), P3 (**C**), and P1 (**D**) as measured by xCELLigence. E/T indicates the specific effector/target cell ratios. (**E**) Sequential microscopic images of the co-culture of CD19NK or CD44NK cells with primary ovarian cancer cells with an E/T ratio of 5:1.

To analyze the killing activity of CAR-NK-92 cells against primary cancer cells, three patient-derived ovarian cancer cell samples (P1-3) were used. These cells were harvested from ascites of one patient at three different time points during chemotherapy. P1 was obtained before paclitaxel treatment, P2 and P3 were collected 20 and 30 days after initiation of paclitaxel treatment, respectively. The cells were characterized by high EPCAM and mesothelin expression (see Figure S3). The ascites cells also showed high CD44 expression, which did not decrease during or after chemotherapy (Figure 3A). We used the xCELLigence analyzer to measure the cytotoxicity of engineered NK cells, as the primary ovarian cancer cells were not labeled with fluorescence markers. Co-incubation of primary cells with CD44NK cells resulted in pronounced and dose-dependent lysis of cancer cells over time (Figure 3B–D). A complete lysis of target cells was already observed in P1 and P3 at an E/T ratio of 5:1, target cells reached baseline CI values after 4 h in P1 and 9 h in P3. A higher E/T ratio (10:1) led to an accelerated lysis of P3 within 4 h. Adding CD44NK cells at the E/T ratio of 1:1 caused a significant CI value reduction within 9 h in P3 (Figure 3C). To verify killing after co-incubation, microscopic images of P3 cells before and 2 h after co-incubation with control NK-92 or CAR-engineered NK cells at the E/T ratio with 5:1 are shown in Figure 3E.

### 3.4. Determination of CAR-NK-92 Activity by IFNγ Quantification

**Figure 4:**
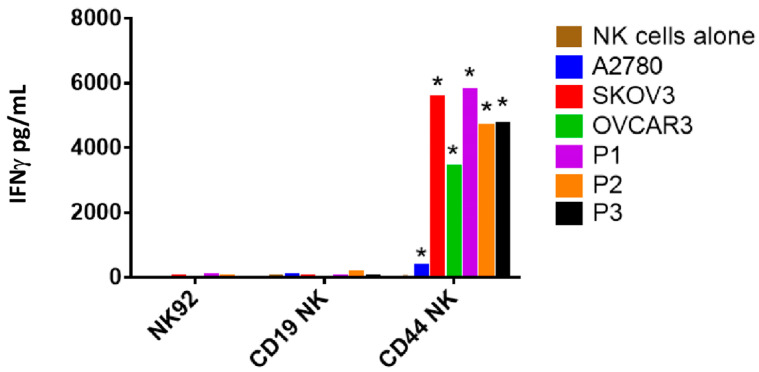
Quantification of IFNγ produced by NK-92 and CAR-NK-92 cells. IFNγ concentration in the cell-free supernatant after co-culture for 24 h with an E/T ratio of 5:1 was measured by ELISA. Values represent the mean ± standard deviation from two separate experiments, each containing three samples. * *p* < 0.05 compared to CD19NK.

To quantify IFNγ secretion, NK-92 and CAR-NK-92 cells were either cultured alone or co-cultured with target cells at an E/T ratio of 5:1. After 24 h, cell-free supernatants were harvested and measured for IFNγ secretion using an ELISA. As shown in Figure 4, NK-92 and CAR-NK-92 cells spontaneously produced negligible or low levels of IFNγ when incubated alone. CD44NK cells were strongly activated by CD44 high expressing SKOV3, OVCAR3 and primary cells, and produced significantly increased IFNγ (*p* < 0.0001). Compared to CD19NK, co-incubation with CD44NK cells resulted in a 174- and 111-fold increase in IFNγ secretion level in P1 and P3, respectively. A five-fold increase in IFNγ secretion level was also shown in co-culture of A2780 cells with CD44NK cells compared to A2780 cells co-cultured with CD19NK cells. This may be due to the very low expression of CD44 on A2780 cells.

### 3.5. Additive Anti-Tumor Activity of CD44NK Cells in Combination with Cisplatin Treatment

**Figure 5:**
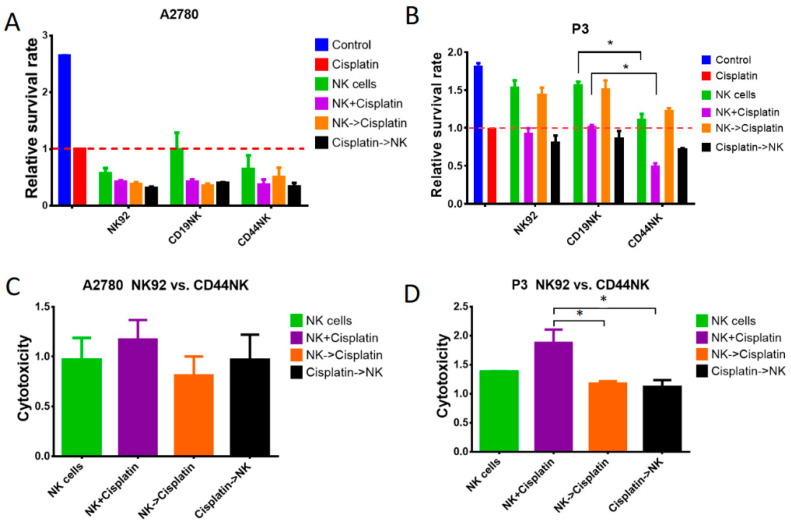
Additive anti-tumor activity of CD44NK cells in combination with cisplatin treatment. A2780 (**A**) and P3 (**B**) cells were treated for 4 days (cisplatin treatment for 72 h, NK-92 or CAR-NK-92 treatment 24 h) with the previously determined IC_50_ dose of cisplatin and an E/T ratio of 2:1. Relative survival rate was calculated by dividing the results obtained from each group by the results of cisplatin monotherapy. Tumor cell loss of A2780 (**C**) and P3 (**D**) was calculated by dividing the results of CD44NK cells by the results of untransduced NK-92 cells for the designated conditions. * indicates a significant difference calculated by one-way ANOVA, *p* < 0.05. Values represent the mean from two separate experiments each containing three samples.

Platinum-based chemotherapy remains the core of primary treatment of advanced-stage ovarian cancer. In an attempt to maximize the benefits of the current therapy, the anti-tumor effect of combinatorial treatment of cisplatin and CAR-NK-92 cells was evaluated. CD44-negative A2780 and CD44-positive P3 cells were used as target cells. Co-incubation was performed for 4 days with NK cells at an E/T ratio of 2:1 and with the previously determined cisplatin IC_50_ dose. For the sequential treatment, co-incubation with NK cells and cisplatin was performed over 24 and 72 h, respectively. Relative survival rates were calculated by dividing the results obtained from each group by the results of cisplatin monotherapy (Figure 5A,B). CAR-NK-92 and untransduced NK-92 cells were still cytotoxic under cisplatin treatment. (Figure 5B). Compared to CD19NK and untransduced NK-92 cells, CD44NK cells significantly enhanced anti-tumor effects when applied as monotherapy (*p* < 0.05) and with simultaneous treatment using cisplatin (*p* < 0.005) (Figure 5B). Notably, this effect was not observed in CD44 negative A2780 cells under all designated conditions (*p* > 0.31) (Figure 5A).

To further evaluate the benefit of incorporating CD44NK cells in the treatment of ovarian cancer, we calculated the relative cytotoxicity of CD44NK cells in relation to the unspecific NK-92 cells to rule out possible unspecific killing effects (Figure 5C,D). As expected, no significant differences were observed in CD44 negative A2780 cells (Figure 5C). CD44NK cells showed significantly higher anti-tumor activity in the simultaneous treatment with cisplatin than in sequential treatment (*p* < 0.05) (Figure 5D). The simultaneous treatment with CD44NK cells and cisplatin also resulted in the lowest survival rate in the P3 primary ovarian cancer sample (*p* < 0.05) (Figure 5B).

## 4. Discussion

In this work, we describe a novel anti-CD44 third-generation CAR directed against ovarian cancer. The increased efficiency of anti-ovarian cancer activity by CD44NK cells led to specific killing activity against ovarian cancer cell lines and patient-derived ovarian cancer cells in various assays. Concurrent therapy with cisplatin increased the killing effect compared to the respective monotherapy or a sequential therapy. Thus, the combination of immunotherapy with cisplatin could be a promising treatment strategy for advanced or relapsed ovarian cancer.

CD44 and its variants represent promising targets for ovarian cancer immunotherapy. The expression of CD44 was shown to be more pronounced in the recurrent and metastatic ovarian cancer tissues, when compared with its primary counterparts. Most ovarian cancer cells are initially chemosensitive. However, there is a population of highly chemoresistant cells with stem cell properties that survives initial therapy [24]. The exact surface markers characterizing ovarian CSCs remain controversial, but CD133, CD44, and CD24 are widely described as ovarian CSC markers in current studies [25]. Importantly, a correlation between the poor outcome of ovarian cancer patients and the expression of these CSC markers has been observed [26,27]. Contrary to other CSC markers, i.e., CD133, CD44 is widely expressed in ovarian cancer cells and was highly expressed in the samples analyzed in this study. Interestingly, several studies found CD44 expression to be associated with CSC-like properties of ovarian cancer cells. For example, ALDH1-bright cells from ovarian cancer cell lines show increased CD44 expression [28] and inhibition of CD44 was shown to inhibit growth of sphere-forming ovarian cancer cells [29]. However, the role of CD44 as a stem cell marker is still controversial and needs to be investigated in further studies.

Independently from its role as a CSC marker, a significant correlation was demonstrated between CD44 expression and disease-free survival and overall survival [27]. It was reported that peritoneal cells produce several extracellular matrix molecules that interact with CD44 [30,31]. CD44 was shown to mediate ovarian cancer cell adhesion to peritoneal mesothelial cells by binding cell surface HA, and thus promotes intraperitoneal ovarian cancer spread [19,20,32]. Downregulation of CD44 expression dramatically decreased the migratory potentials and invasiveness of ovarian cancer cells in vitro and suppressed tumor growth and peritoneal dissemination of human ovarian cancer xenograft in nude mice [27,33]. Interestingly, a recent study demonstrated that ovarian cancer-derived exosomes could transfer CD44 to human peritoneal mesothelial cells (HPMCs) and induce morphologic change in HPMCs to a mesenchymal, spindle phenotype. These exosomes increased CD44 expression in HPMCs, which facilitated cancer invasion by inducing the HPMCs to secrete matrix metalloproteinase-9 (MMP9) [34]. Furthermore, CD44 expression was shown to be significantly higher in the paclitaxel-resistant ovarian cell lines than in the drug-sensitive parental cell lines. Overexpression of CD44 was found in relapsed/recurrent tumors in a xenograft mouse model treated with paclitaxel [27]. CD44 targeted therapy was shown to effectively inhibit ovarian cancer dissemination, abrogate ascites, and prolong survival time [35,36]. All of these pieces of evidence suggest that developing new strategies to target CD44 in ovarian cancer may prevent disease recurrence, metastasis, and chemoresistance.

CAR-based immunotherapy has shown outstanding results in hematological malignancies [37]. Ovarian cancer is an immunogenic tumor with several tumor-specific antigens, which makes it an excellent target for CAR therapy. Sun et al. developed a humanized HER2 CAR containing chA21 scFv and T-cell intracellular signaling chains made up of CD28 and CD3ζ. They demonstrated that anti-HER2 CAR T cells were able to recognize and kill ovarian cancer cells ex vivo [38]. CAR-modified MUC-CD-targeted T cells infused through either intravenous or intraperitoneal injection showed either delayed progression or fully eradicated disease in SCID-Beige mice bearing orthotopic human MUC-CD-positive ovarian carcinoma tumors [9]. However, an early phase I study using anti-FRα CAR T cells in ovarian cancer patients reported that tumor burden was not significantly reduced in any patient. CAR T cells were present in the circulation in large numbers for the first 2 days after transfer, but rapidly declined to barely detectable levels one month later in most patients [39]. Although robust anti-tumor activity of anti-FRα CAR T cells was shown in vitro, they were unable to show tumor regression in clinical settings in patients due to their inability to persist in the tumor microenviroment. To overcome this limitation, Song and colleagues developed a new anti-FRα CAR in combination with a 4-1BB co-stimulatory motif and demonstrated that 4-1BB improved CAR T cell persistence in vivo [10]. Carpenito et al. equipped T cells with a third-generation mesothelin-specific CAR consisting of CD28 and 4-1BB as co-stimulators, and transferred them intratumorally and intravenously into mice engrafted with pre-established tumors. After infusion of those CAR T cells, tumor burden was reduced, and complete eradication of the tumors was observed in some cases [40]. However, a CAR with mouse origin scFvs has limited anti-tumor effect due to transgene immunogenicity. Therefore, a second-generation fully human anti-mesothelin CAR (P4 CAR) was developed. Primary human T cells expressing P4 CAR efficiently killed mesothelin-expressing tumors in vitro and in vivo [8]. A phase I trial for infusion of anti-mesothelin CAR T cells in patients with recurrent serous ovarian cancer was shown to be feasible and safe [41].

In contrast to other studies that used T cells in CAR therapy for ovarian cancer [8,9,38,39,40,41,42], we chose NK-92 as effector cells in this study. NK-92 cells were established from a patient with non-Hodgkin’s lymphoma and their safety and anti-tumor capability were shown in clinical trials [43,44]. NK-92 cells were shown to be generally safe with moderate and transient toxicities in advanced cancer patients, and large-scale expansion of these cells is feasible [44]. Importantly, NK-92 cells express a relatively large number of activating receptors and lack most of the killer inhibitory receptors (KIRs) that are normally expressed on NK cells. Furthermore, NK-92 express high levels of molecules involved in the perforin-granzyme cytolytic pathway and additional cytotoxic effector molecules, indicating the potential of alternative anti-tumor mechanisms [45]. This confers NK-92 cells with superior cytotoxicity against a broad spectrum of tumor targets. Kloess and colleagues compared the function of CD123-CAR-expressing NK-92 cells and primary human donor NK (dNK) cells. They demonstrated that CAR-NK-92 cells had significantly stronger cytotoxic activity against leukemia cells as compared to CAR-dNK cells. The cytokine secretion profiles of CAR-NK-92 and CAR-dNK cells were shown to be very different [46]. However, it is important to note that CAR-NK-92 cells also exhibited significantly higher potential for adverse side effects against non-target cells [46]. Notably, NK-92 cells can be easily expanded in cultures with short doubling times to generate potent clinical-grade NK-92 effectors [47] and require only minimal manipulation without the need for cell-selection procedures. Thus, NK-92 cells offer an attractive platform for future study of novel NK-cell-based therapy.

In this study, we chose CD44 as a promising target and developed a novel third-generation anti-CD44 CAR incorporating a fully human anti-CD44 scFv linked to CD28 and 4-1BB as co-stimulators and a CD3ζ signaling domain. NK-92 cells equipped with the anti-CD44 CAR exhibited potent cytotoxic activity against CD44-positive ovarian cancer cell lines and primary ovarian cancer cells. Among these target cancer cells, A2780, OVCAR3, P2 and P3 cells also appeared to be sensitive to nonspecific cytotoxicity of CD19NK and NK-92 cells. However, CAR-restricted killing induced by CD44NK occurred more rapidly and more potently in CD44-positive OVCAR3, P2 and P3 cells. In parallel, the measurement of NK cell activation by IFNγ secretion assays confirmed these observations. Meanwhile, this significantly increased CAR-restricted cytotoxicity of CD44NK was hardly observed in CD44-low-expressing A2780 cells. Interestingly, we detected a slightly increased IFNγ level in co-culture of A2780 cells with CD44NK cells compared to that with CD19NK cells. This may be due to the very low expression of CD44 on A2780 cells, and the death of those cells was not detected by fluorescent microscopy or Fluoroskan measurements. Notably, P1 cells exhibited high resistance to nonspecific cytotoxicity of CD19NK and NK-92 cells even with a higher E/T ratio of 10:1 (data not shown), but these primary cancer cells strongly activated CD44NK cells and were largely eliminated by CD44NK cells with a lower E/T ratio of 5:1. In this study, we tested the killing effect of CD44NK cells against three primary samples isolated from one patient at different time points. Therefore, we cannot predict cytotoxic effects against all ovarian cancer cells in general. Since CD44NK cells showed activity against all tested cell lines and primary cells that express CD44, we assume that the CD44NK cells will also be effective against other ovarian cancer cells that express CD44. However, we also know that the cytotoxic activity of CAR-T or CAR-NK cells depends on susceptibility of the individual cells as shown in Figure 3. Thus, each patient has to be screened for CD44 expression before CAR therapy can be considered.

A previous study indicated that carboplatin induced CD44 expressing ovarian cancer cells to produce HA, which can contribute to chemoresistance by regulating ATP binding cassette transporter expression [48]. This highlights the importance of combinatorial treatment of chemotherapy and CD44-targeted therapy in ovarian cancer. To investigate the feasibility of combinatorial treatment of cytoreductive chemotherapy and specific elimination of CD44-positive highly chemoresistant tumor cells by CD44NK cells, we tested the anti-tumor activity of cisplatin and CAR-NK-92 cells in monotherapy, simultaneous, and sequential treatment. To mimic clinical settings and reduce the nonspecific cytotoxicity of NK cells, we used a low E/T ratio of 2:1. Compared to cisplatin monotherapy, simultaneous treatment with CD44NK cells and cisplatin resulted in a significant additive anti-tumor effect on P3 cells (27.1% more cell reduction). In contrast, simultaneous treatment with control NK cells and cisplatin did not show any additive effect in P3 primary ovarian cancer cells.

However, CAR-based therapy for solid tumors is faced with many challenges. In hematological malignancies, circulating CAR-engineered effector cells in the bloodstream have already reached the majority of their target cancer cells. In solid tumors, there are multiple barriers that hinder therapeutically sufficient tumor infiltration by CAR effector cells (reviewed in [49]). Regional CAR effector cell administration is one method to facilitate NK cell interaction with the tumor and potentially reduce off-tumor toxicity. A study of intraperitoneal (i.p.) injection of pan-ErbB/IL-4 CAR T cells targeting patient-derived malignant pleural mesotheliomas xenografts in SCID mice showed tumor regression or cure in all mice [50]. Katz and colleagues showed that i.p. delivery of CAR T cells resulted in superior protection against peritoneal tumors, when compared with systemically infused CAR T cells. I.p. infusion also provided prolonged protection against i.p. tumor relapse and demonstrated an increased effector memory phenotype over time [51]. Further in vivo studies are required to evaluate the efficacy and safety of CD44NK treatment.

CD44 is also widely distributed in normal tissues, e.g., central nervous system, lung, and hematopoietic cells [52]. The potential toxicity of CD44NK cells on normal tissues should be investigated in further studies. Strategies to prevent possible side effects, e.g., by using bispecific CARs, are already being evaluated in our lab.

In summary, we developed a novel third-generation CAR against CD44. The CAR-restricted killing effect of CD44NK cells was demonstrated in vitro. Additionally, we investigated combinatorial treatment strategies of CD44NK cells and cisplatin therapy and showed that CD44NK cells retained cytotoxicity during cisplatin incubation. The most potent anti-tumor effect was achieved by simultaneous treatment with CD44NK cells and cisplatin. This study will be the basis for further in vivo studies and future clinical developments.

## Data Availability

Data is contained within the article and Supplementary Material.

## Supplementary Materials

The following are available online at https://www.mdpi.com/article/10.3390/biomedicines9101339/s1, Figure S1. CD44NK effectively kill A2780 cells that were modified to express CD44, Figure S2. CD44-specific killing capacity of CD44NK cells. CD44NK cells were co-cultured with A2780 eGFP cells, A2780 CD44iGFP cells or OVCAR3 eGFP cells at an effector to target (E:T) ratio of 5:1, Figure S3. Flow cytometric analyses of EPCAM and Mesothelin expression levels on the three different primary ovarian cancer cell samples.

## References

[1] Bray F., Ferlay J., Soerjomataram I., Siegel R.L., Torre L.A., Jemal A. (2018). Global cancer statistics 2018: GLOBOCAN estimates of incidence and mortality worldwide for 36 cancers in 185 countries. CA Cancer J. Clin..

[2] Heintz A., Odicino F., Maisonneuve P., A Quinn M., Benedet J.L., Creasman W.T., Ngan H.Y.S., Pecorelli S., Beller U. (2006). Carcinoma of the Ovary. Int. J. Gynecol. Obstet..

[3] Galon J., Angell H.K., Bedognetti D., Marincola F.M. (2013). The Continuum of Cancer Immunosurveillance: Prognostic, Predictive, and Mechanistic Signatures. Immunity.

[4] Zhang L., Conejo-Garcia J.R., Katsaros D., Gimotty P.A., Massobrio M., Regnani G., Makrigiannakis A., Gray H., Schlienger K., Liebman M.N. (2003). Intratumoral T Cells, Recurrence, and Survival in Epithelial Ovarian Cancer. N. Engl. J. Med..

[5] Sato E., Olson S.H., Ahn J., Bundy B., Nishikawa H., Qian F., Jungbluth A.A., Frosina D., Gnjatic S., Ambrosone C. (2005). Intraepithelial CD8+ tumor-infiltrating lymphocytes and a high CD8+/regulatory T cell ratio are associated with favorable prognosis in ovarian cancer. Proc. Natl. Acad. Sci. USA.

[6] Hwang W.-T., Adams S.F., Tahirovic E., Hagemann I., Coukos G. (2012). Prognostic significance of tumor-infiltrating T cells in ovarian cancer: A meta-analysis. Gynecol. Oncol..

[7] Li J., Wang J., Chen R., Bai Y., Lu X. (2017). The prognostic value of tumor-infiltrating T lymphocytes in ovarian cancer. Oncotarget.

[8] Lanitis E., Poussin M., Hagemann I., Coukos G., Sandaltzopoulos R., Scholler N., Powell D.J. (2012). Redirected Antitumor Activity of Primary Human Lymphocytes Transduced with a Fully Human Anti-mesothelin Chimeric Receptor. Mol. Ther..

[9] Chekmasova A.A., Rao T.D., Nikhamin Y., Park K., Levine D.A., Spriggs D.R., Brentjens R.J. (2010). Successful Eradication of Established Peritoneal Ovarian Tumors in SCID-Beige Mice following Adoptive Transfer of T Cells Genetically Targeted to the MUC16 Antigen. Clin. Cancer Res..

[10] Song D.-G., Ye Q., Carpenito C., Poussin M., Wang L.-P., Ji C., Figini M., June C.H., Coukos G., Powell D.J. (2011). In Vivo Persistence, Tumor Localization, and Antitumor Activity of CAR-Engineered T Cells Is Enhanced by Costimulatory Signaling through CD137 (4-1BB). Cancer Res..

[11] Klapdor R., Wang S., Hacker U., Büning H., Morgan M., Dörk T., Hillemanns P., Schambach A. (2017). Improved Killing of Ovarian Cancer Stem Cells by Combining a Novel Chimeric Antigen Receptor–Based Immunotherapy and Chemotherapy. Hum. Gene Ther..

[12] Foster R., Buckanovich R.J., Rueda B.R. (2013). Ovarian cancer stem cells: Working towards the root of stemness. Cancer Lett..

[13] Latifi A., Abubaker K., Castrechini N., Ward A., Liongue C., Dobill F., Kumar J., Thompson E.W., Quinn M., Findlay J.K. (2011). Cisplatin treatment of primary and metastatic epithelial ovarian carcinomas generates residual cells with mesenchymal stem cell-like profile. J. Cell. Biochem..

[14] Takaishi S., Okumura T., Tu S., Wang S.S., Shibata W., Vigneshwaran R., Gordon S.A., Shimada Y., Wang T.C. (2009). Identification of Gastric Cancer Stem Cells Using the Cell Surface Marker CD44. STEM CELLS.

[15] Prince M.E., Sivanandan R., Kaczorowski A., Wolf G.T., Kaplan M.J., Dalerba P., Weissman I.L., Clarke M.F., Ailles L.E. (2007). Identification of a subpopulation of cells with cancer stem cell properties in head and neck squamous cell carcinoma. Proc. Natl. Acad. Sci. USA.

[16] Zhang S., Balch C., Chan M., Lai H.-C., Matei D., Schilder J.M., Yan P.S., Huang T.H.-M., Nephew K.P. (2008). Identification and Characterization of Ovarian Cancer-Initiating Cells from Primary Human Tumors. Cancer Res..

[17] Orian-Rousseau V. (2010). CD44, a therapeutic target for metastasising tumours. Eur. J. Cancer.

[18] Zöller M. (2011). CD44: Can a cancer-initiating cell profit from an abundantly expressed molecule?. Nat. Rev. Cancer.

[19] Lessan K., Aguiar D.J., Oegema T., Siebenson L., Skubitz A.P. (1999). CD44 and β1 Integrin Mediate Ovarian Carcinoma Cell Adhesion to Peritoneal Mesothelial Cells. Am. J. Pathol..

[20] A Cannistra S., Kansas G.S., Niloff J., Defranzo B., Kim Y., Ottensmeier C. (1993). Binding of ovarian cancer cells to peritoneal mesothelium in vitro is partly mediated by CD44H. Cancer Res..

[21] Runnels H.A., Weber G.L., Min J., Kudlacz E.M., Zobel J.F., Donovan C.B., Thiede M.A., Zhang J., Alpert R.B., Salafia M.A. (2010). PF-03475952: A potent and neutralizing fully human anti-CD44 antibody for therapeutic applications in inflammatory diseases. Adv. Ther..

[22] Suerth J.D., Morgan M.A., Kloess S., Heckl D., Neudörfl C., Falk C.S., Koehl U., Schambach A. (2016). Efficient generation of gene-modified human natural killer cells via alpharetroviral vectors. J. Mol. Med..

[23] Dull T., Zufferey R., Kelly M., Mandel R.J., Nguyen M., Trono D., Naldini L. (1998). A Third-Generation Lentivirus Vector with a Conditional Packaging System. J. Virol..

[24] Albini A., Bruno A., Gallo C., Pajardi G.E., Noonan U.M., Dallaglio K. (2015). Cancer stem cells and the tumor microenvironment: Interplay in tumor heterogeneity. Connect. Tissue Res..

[25] Garson K., Vanderhyden B.C. (2015). Epithelial ovarian cancer stem cells: Underlying complexity of a simple paradigm. Reproduction.

[26] Steffensen K.D., Alvero A.B., Yang Y., Waldstrøm M., Hui P., Holmberg J.C., Silasi D.-A., Jakobsen A., Rutherford T., Mor G. (2011). Prevalence of Epithelial Ovarian Cancer Stem Cells Correlates with Recurrence in Early-Stage Ovarian Cancer. J. Oncol..

[27] Gao Y., Foster R., Yang X., Feng Y., Shen J.K., Mankin H.J., Hornicek F.J., Amiji M.M., Duan Z. (2015). Up-regulation of CD44 in the development of metastasis, recurrence and drug resistance of ovarian cancer. Oncotarget.

[28] Wang Y.-C., Yo Y.-T., Lee H.-Y., Liao Y.-P., Chao T.-K., Su P.-H., Lai H.-C. (2012). ALDH1-Bright Epithelial Ovarian Cancer Cells Are Associated with CD44 Expression, Drug Resistance, and Poor Clinical Outcome. Am. J. Pathol..

[29] Du Y.-R., Chen Y., Gao Y., Niu X.-L., Li Y.-J., Deng W.-M. (2013). Effects and Mechanisms of Anti-CD44 Monoclonal Antibody A3D8 on Proliferation and Apoptosis of Sphere-Forming Cells with Stemness From Human Ovarian Cancer. Int. J. Gynecol. Cancer.

[30] Dimitroff C.J., Lee J.Y., Fuhlbrigge R.C., Sackstein R. (2000). A distinct glycoform of CD44 is an L-selectin ligand on human hematopoietic cells. Proc. Natl. Acad. Sci. USA.

[31] Dimitroff C.J., Lee J.Y., Rafii S., Fuhlbrigge R.C., Sackstein R. (2001). Cd44 Is a Major E-Selectin Ligand on Human Hematopoietic Progenitor Cells. J. Cell Biol..

[32] Carpenter P.M., Dao A.V. (2003). The role of hyaluronan in mesothelium-induced motility of ovarian carcinoma cells. Anticancer Res..

[33] Cheng W., Liu T., Wan X., Gao Y., Wang H. (2012). MicroRNA-199a targets CD44 to suppress the tumorigenicity and multidrug resistance of ovarian cancer-initiating cells. FEBS J..

[34] Nakamura K., Sawada K., Kinose Y., Yoshimura A., Toda A., Nakatsuka E., Hashimoto K., Mabuchi S., Morishige K.-I., Kurachi H. (2016). Exosomes Promote Ovarian Cancer Cell Invasion through Transfer of CD44 to Peritoneal Mesothelial Cells. Mol. Cancer Res..

[35] De Stefano I., Battaglia A., Zannoni G.F., Prisco M.G., Fattorossi A., Travaglia D., Baroni S., Renier D., Scambia G., Ferlini C. (2011). Hyaluronic acid–paclitaxel: Effects of intraperitoneal administration against CD44(+) human ovarian cancer xenografts. Cancer Chemother. Pharmacol..

[36] Lee S.J., Ghosh S.C., Han H.D., Stone R.L., Bottsford-Miller J., Shen D.Y., Auzenne E.J., Lopez-Araujo A., Lu C., Nishimura M. (2012). Metronomic Activity of CD44-Targeted Hyaluronic Acid-Paclitaxel in Ovarian Carcinoma. Clin. Cancer Res..

[37] Park J.H., Geyer M.B., Brentjens R.J. (2016). CD19-targeted CAR T-cell therapeutics for hematologic malignancies: Interpreting clinical outcomes to date. Blood.

[38] Sun M., Shi H., Liu C., Liu J., Liu X., Sun Y. (2014). Construction and evaluation of a novel humanized HER2-specific chimeric receptor. Breast Cancer Res..

[39] Kershaw M.H., Westwood J.A., Parker L.L., Wang G., Eshhar Z., Mavroukakis S.A., White D.E., Wunderlich J.R., Canevari S., Rogers-Freezer L. (2006). A Phase I Study on Adoptive Immunotherapy Using Gene-Modified T Cells for Ovarian Cancer. Clin. Cancer Res..

[40] Carpenito C., Milone M.C., Hassan R., Simonet J.C., Lakhal M., Suhoski M.M., Varela-Rohena A., Haines K.M., Heitjan D.F., Albelda S.M. (2009). Control of large, established tumor xenografts with genetically retargeted human T cells containing CD28 and CD137 domains. Proc. Natl. Acad. Sci. USA.

[41] Tanyi J.L., Haas A.R., Beatty G.L., Stashwick C.J., O’Hara M., Morgan M.A., Porter D.L., Melenhorst J.J., Plesa G., Lacey S.F. (2016). Anti-mesothelin chimeric antigen receptor T cells in patients with epithelial ovarian cancer. J. Clin. Oncol..

[42] Koneru M., Purdon T., Spriggs D., Koneru S., Brentjens R.J. (2015). IL-12 secreting tumor-targeted chimeric antigen receptor T cells eradicate ovarian tumorsin vivo. OncoImmunology.

[43] Tonn T., Becker S., Esser R., Schwabe D., Seifried E. (2001). Cellular Immunotherapy of Malignancies Using the Clonal Natural Killer Cell Line NK-92. J. Hematother..

[44] Arai S., Meagher R., Swearingen M., Myint H., Rich E., Martinson J., Klingemann H. (2008). Infusion of the allogeneic cell line NK-92 in patients with advanced renal cell cancer or melanoma: A phase I trial. Cytotherapy.

[45] Maki G., Klingemann H.-G., Martinson J.A., Tam Y.K. (2001). Factors Regulating the Cytotoxic Activity of the Human Natural Killer Cell Line, NK-92. J. Hematother..

[46] Kloess S., Oberschmidt O., Dahlke J., Vu X.-K., Neudoerfl C., Kloos A., Gardlowski T., Matthies N., Heuser M., Meyer J. (2019). Preclinical Assessment of Suitable Natural Killer Cell Sources for Chimeric Antigen Receptor Natural Killer–Based “Off-the-Shelf” Acute Myeloid Leukemia Immunotherapies. Hum. Gene Ther..

[47] Tarn Y., Martinson J., Doligosa K., Klingernann H.-G. (2003). Ex vivo expansion of the highly cytotoxic human natural killer cell line NK-92 under current good manufacturing practice conditions for clinical adoptive cellular immunotherapy. Cytotherapy.

[48] Ricciardelli C., Ween M.P., A Lokman N., A Tan I., E Pyragius C., Oehler M.K. (2013). Chemotherapy-induced hyaluronan production: A novel chemoresistance mechanism in ovarian cancer. BMC Cancer.

[49] Vignali D., Kallikourdis M. (2017). Improving homing in T cell therapy. Cytokine Growth Factor Rev..

[50] Klampatsa A., Achkova D.Y., Davies D.M., Parente-Pereira A.C., Woodman N., Rosekilly J., Osborne G., Thayaparan T., Bille A., Sheaf M. (2017). Intracavitary ‘T4 immunotherapy’ of malignant mesothelioma using pan-ErbB re-targeted CAR T-cells. Cancer Lett..

[51] Katz S., Point G.R., Cunetta M., Thorn M., Guha P., Espat N.J., Boutros C., Hanna N., Junghans R.P. (2016). Regional CAR-T cell infusions for peritoneal carcinomatosis are superior to systemic delivery. Cancer Gene Ther..

[52] Fox S., Fawcett J., Jackson D.G., Collins I., Gatter K.C., Harris A., Gearing A., Simmons D.L. (1994). Normal human tissues, in addition to some tumors, express multiple different CD44 isoforms. Cancer Res..

