# Supplementary material for: NK Cell-Mediated Eradication of Ovarian Cancer Cells with a Novel Chimeric Antigen Receptor Directed against CD44
Source: Biomedicines. 2021 Sep 28;9(10):1339. doi: 10.3390/biomedicines9101339 (PMC8533227; doi:10.3390/biomedicines9101339)
Supplement: Supplementary file 1 [file biomedicines-09-01339-s001.zip › biomedicines-1378466-supplementary.pdf]

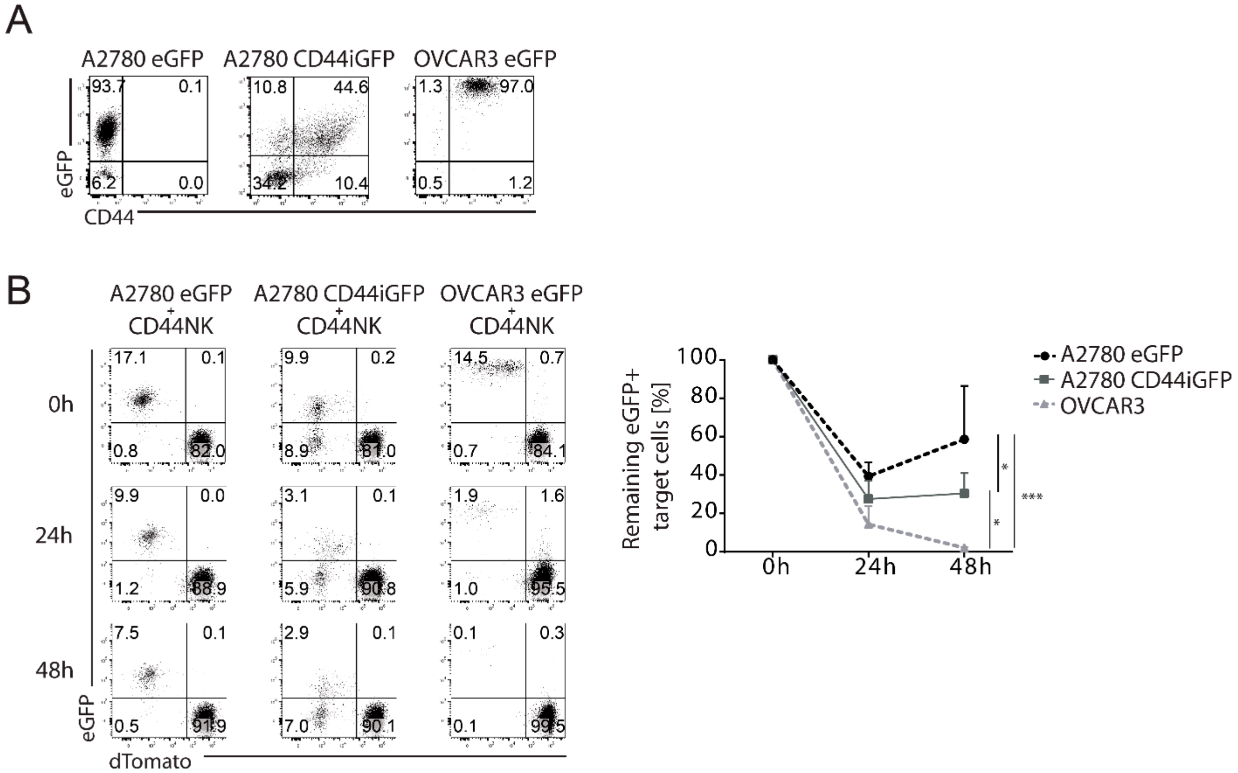

Figure S1. CD44NK effectively kill A2780 cells that were modified to express CD44. (A) CD44 expression in A2780 eGFP cells (negative control; CD44-), A2780 cells modified with a bicistronic lentiviral vector to express CD44 and eGFP via an internal ribosomal entry site (A2780 CD44iGFP cells) (CD44+) and OVCAR3 eGFP cells (positive control; CD44+). (B) Cytotoxic capacity of CD44NK cells against CD44+ and CD44- target cells. CD44NK cells were co-cultured with A2780 eGFP cells, A2780 CD44iGFP cells or OVCAR3 eGFP cells at an effector to target (E:T) ratio of 5:1. Remaining target cells (eGFP+) and CD44NK cells (dTomato) were quantified via flow cytometric analysis after 24h and 48h. The percentage of eGFP+ target cells at the beginning of the experiment (0h) was set as 100% and the percentage of eGFP+ target cells remaining at 24h and 48h were calculated respectively. Results are from three independent experiments that were each accomplished in duplicates. Indicated significance was determined for the 48h time point by two-way ANOVA with Tukey´s multiple comparison test; *** $p \leq 0.001$; ** $p \leq 0.01$; * $p \leq 0.05$. Mean values ±SD are shown.

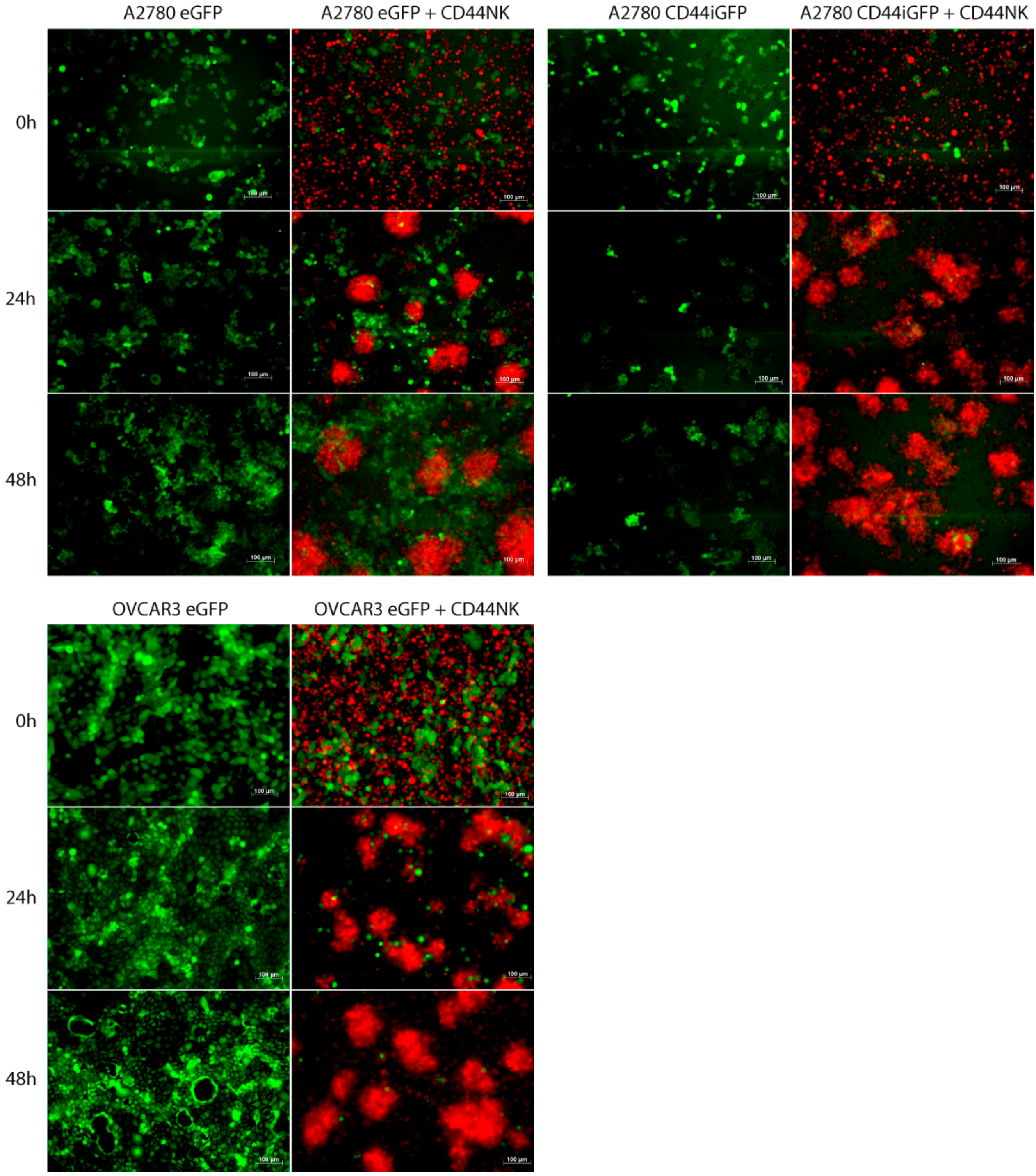

Figure S2. CD44-specific killing capacity of CD44NK cells. CD44NK cells were co-cultured with A2780 eGFP cells, A2780 CD44iGFP cells or OVCAR3 eGFP cells at an effector to target (E:T) ratio of 5:1. Co-cultures were observed via fluorescence microscopy after 0h, 24h and 48h. CD44NK cells are visualized as red/dTomato+, and target cells as green/eGFP+. These exemplary pictures correspond to the experiments shown in Figure S1.

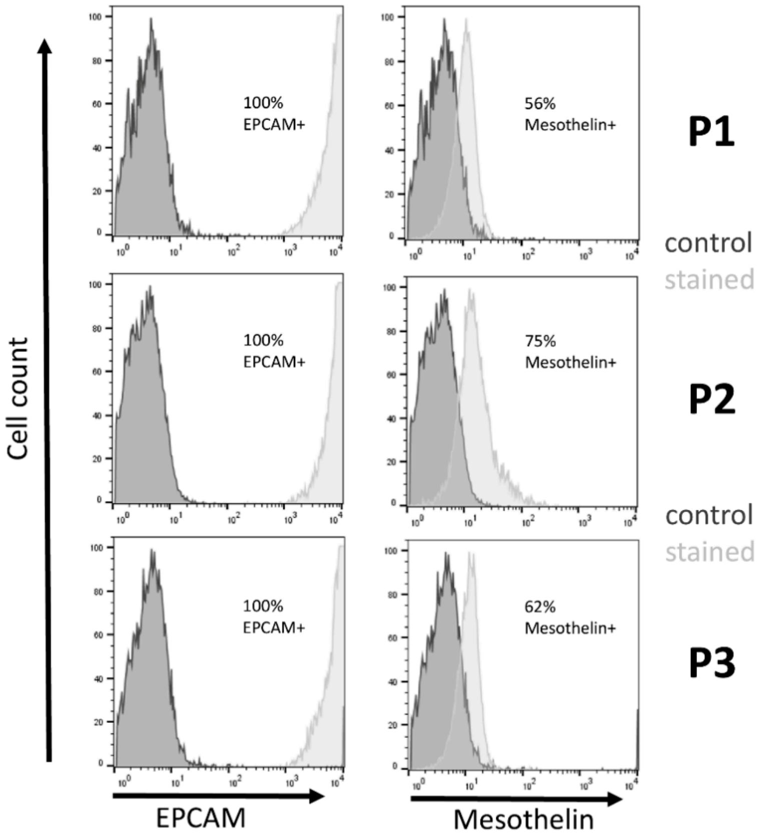

Figure S3. Flow cytometric analyses of EPCAM and Mesothelin expression levels on the three different primary ovarian cancer cell samples. Control refers to unstained sample.
